# Supplementary material for: Growth behavior and glyphosate resistance level in 10 populations of Echinochloa colona in Australia
Source: PLoS One. 2020 Jan 14;15(1):e0221382. doi: 10.1371/journal.pone.0221382 (PMC6959982; doi:10.1371/journal.pone.0221382)

**Probit Analysis BY17/49**

| **Notes** | | |
| --- | --- | --- |
| Comments | |  |
| Input | Data | C:\Users\uqgmahaj\OneDrive - The University of Queensland\Desktop\BY22.sav |
|  | Active Dataset | DataSet0 |
|  | Filter | <none> |
|  | Weight | <none> |
|  | Split File | <none> |
|  | N of Rows in Working Data File | 5 |
| Missing Value Handling | Definition of Missing | User-defined missing values are treated as missing. |
|  | Cases Used | Statistics are based on all cases with valid data for all variables in the model. |
| Syntax | | PROBIT VAR00003 OF VAR00002 WITH VAR00001  /LOG 10  /MODEL PROBIT  /PRINT FREQ CI  /CRITERIA P(0.15) ITERATE(20) STEPLIMIT(.1). |
| Resources | Processor Time | 00:00:00.17 |
|  | Elapsed Time | 00:00:00.18 |

[DataSet0] C:\Users\uqgmahaj\OneDrive - The University of Queensland\Desktop\BY22.sav

| **Data Information** | | |
| --- | --- | --- |
|  | | N of Cases |
| Valid | | 4 |
| Rejected | Missing | 0 |
|  | LOG Transform Cannot be Done | 0 |
|  | Number of Responses > Number of Subjects | 0 |
| Control Group | | 1 |

| **Convergence Information** | | |
| --- | --- | --- |
|  | Number of Iterations | Optimal Solution Found |
| PROBIT | 10 | Yes |

| **Parameter Estimates** | | | | | | | |
| --- | --- | --- | --- | --- | --- | --- | --- |
|  | Parameter | Estimate | Std. Error | Z | Sig. | 95% Confidence Interval | |
|  |  |  |  |  |  | Lower Bound | Upper Bound |
| PROBIT^a^ | VAR00001 | 1.769 | .218 | 8.123 | .000 | 1.342 | 2.196 |
|  | Intercept | -4.746 | .634 | -7.485 | .000 | -5.380 | -4.112 |
| a. PROBIT model: PROBIT(p) = Intercept + BX (Covariates X are transformed using the base 10.000 logarithm.) | | | | | | | |

| **Chi-Square Tests** | | | | |
| --- | --- | --- | --- | --- |
|  | | Chi-Square | df^b^ | Sig. |
| PROBIT | Pearson Goodness-of-Fit Test | 1.153 | 2 | .562^a^ |
| a. Since the significance level is greater than .150, no heterogeneity factor is used in the calculation of confidence limits. | | | | |
| b. Statistics based on individual cases differ from statistics based on aggregated cases. | | | | |

| **Cell Counts and Residuals** | | | | | | | |
| --- | --- | --- | --- | --- | --- | --- | --- |
|  | Number | VAR00001 | Number of Subjects | Observed Responses | Expected Responses | Residual | Probability |
| PROBIT | 1 | 2.512 | 100 | 40 | 38.106 | 1.894 | .381 |
|  | 2 | 2.813 | 100 | 55 | 59.086 | -4.086 | .591 |
|  | 3 | 3.114 | 100 | 80 | 77.704 | 2.296 | .777 |
|  | 4 | 3.415 | 100 | 90 | 90.229 | -.229 | .902 |

| **Confidence Limits** | | | | | | | |
| --- | --- | --- | --- | --- | --- | --- | --- |
|  | Probability | 95% Confidence Limits for VAR00001 | | | 95% Confidence Limits for log(VAR00001)^a^ | | |
|  |  | Estimate | Lower Bound | Upper Bound | Estimate | Lower Bound | Upper Bound |
| PROBIT | .010 | 23.324 | 7.489 | 47.037 | 1.368 | .874 | 1.672 |
|  | .020 | 33.260 | 11.931 | 62.728 | 1.522 | 1.077 | 1.797 |
|  | .030 | 41.658 | 16.029 | 75.316 | 1.620 | 1.205 | 1.877 |
|  | .040 | 49.347 | 20.013 | 86.438 | 1.693 | 1.301 | 1.937 |
|  | .050 | 56.636 | 23.970 | 96.696 | 1.753 | 1.380 | 1.985 |
|  | .060 | 63.682 | 27.946 | 106.392 | 1.804 | 1.446 | 2.027 |
|  | .070 | 70.578 | 31.968 | 115.699 | 1.849 | 1.505 | 2.063 |
|  | .080 | 77.384 | 36.056 | 124.730 | 1.889 | 1.557 | 2.096 |
|  | .090 | 84.142 | 40.224 | 133.564 | 1.925 | 1.604 | 2.126 |
|  | .100 | 90.884 | 44.482 | 142.256 | 1.958 | 1.648 | 2.153 |
|  | .150 | 125.044 | 67.418 | 184.830 | 2.097 | 1.829 | 2.267 |
|  | .200 | 161.140 | 93.708 | 227.859 | 2.207 | 1.972 | 2.358 |
|  | .250 | 200.305 | 124.139 | 273.019 | 2.302 | 2.094 | 2.436 |
|  | .300 | 243.526 | 159.576 | 321.614 | 2.387 | 2.203 | 2.507 |
|  | .350 | 291.860 | 201.038 | 374.978 | 2.465 | 2.303 | 2.574 |
|  | .400 | 346.566 | 249.755 | 434.729 | 2.540 | 2.398 | 2.638 |
|  | .450 | 409.236 | 307.210 | 503.035 | 2.612 | 2.487 | 2.702 |
|  | .500 | 481.967 | 375.166 | 583.016 | 2.683 | 2.574 | 2.766 |
|  | .550 | 567.624 | 455.665 | 679.406 | 2.754 | 2.659 | 2.832 |
|  | .600 | 670.268 | 551.047 | 799.627 | 2.826 | 2.741 | 2.903 |
|  | .650 | 795.903 | 664.203 | 955.486 | 2.901 | 2.822 | 2.980 |
|  | .700 | 953.872 | 799.560 | 1165.885 | 2.979 | 2.903 | 3.067 |
|  | .750 | 1159.695 | 965.365 | 1462.227 | 3.064 | 2.985 | 3.165 |
|  | .800 | 1441.559 | 1177.998 | 1902.050 | 3.159 | 3.071 | 3.279 |
|  | .850 | 1857.678 | 1471.816 | 2608.599 | 3.269 | 3.168 | 3.416 |
|  | .900 | 2555.932 | 1931.570 | 3914.199 | 3.408 | 3.286 | 3.593 |
|  | .910 | 2760.707 | 2060.809 | 4321.117 | 3.441 | 3.314 | 3.636 |
|  | .920 | 3001.801 | 2210.393 | 4812.643 | 3.477 | 3.344 | 3.682 |
|  | .930 | 3291.270 | 2386.721 | 5419.487 | 3.517 | 3.378 | 3.734 |
|  | .940 | 3647.680 | 2599.514 | 6190.067 | 3.562 | 3.415 | 3.792 |
|  | .950 | 4101.512 | 2864.479 | 7205.981 | 3.613 | 3.457 | 3.858 |
|  | .960 | 4707.354 | 3209.248 | 8617.863 | 3.673 | 3.506 | 3.935 |
|  | .970 | 5576.143 | 3688.762 | 10743.137 | 3.746 | 3.567 | 4.031 |
|  | .980 | 6984.174 | 4436.143 | 14409.826 | 3.844 | 3.647 | 4.159 |
|  | .990 | 9959.334 | 5927.022 | 22914.689 | 3.998 | 3.773 | 4.360 |
| a. Logarithm base = 10. | | | | | | | |


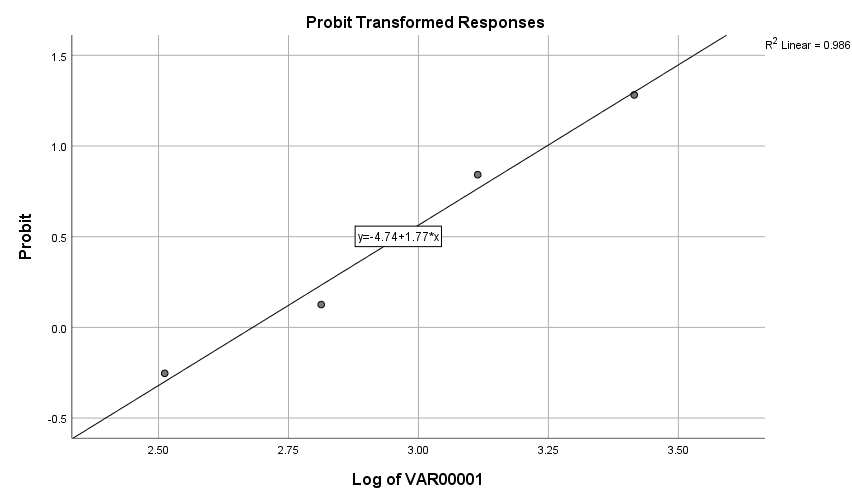


**Probit Analysis 17/37**

| **Data Information** | | |
| --- | --- | --- |
|  | | N of Cases |
| Valid | | 4 |
| Rejected | Missing | 0 |
|  | LOG Transform Cannot be Done | 0 |
|  | Number of Responses > Number of Subjects | 0 |
| Control Group | | 1 |

| **Convergence Information** | | |
| --- | --- | --- |
|  | Number of Iterations | Optimal Solution Found |
| PROBIT | 15 | Yes |

| **Parameter Estimates** | | | | | | | |
| --- | --- | --- | --- | --- | --- | --- | --- |
|  | Parameter | Estimate | Std. Error | Z | Sig. | 95% Confidence Interval | |
|  |  |  |  |  |  | Lower Bound | Upper Bound |
| PROBIT^a^ | VAR00001 | 2.503 | .504 | 4.964 | .000 | 1.515 | 3.491 |
|  | Intercept | -5.523 | 1.351 | -4.088 | .000 | -6.874 | -4.172 |
| a. PROBIT model: PROBIT(p) = Intercept + BX (Covariates X are transformed using the base 10.000 logarithm.) | | | | | | | |

| **Chi-Square Tests** | | | | |
| --- | --- | --- | --- | --- |
|  | | Chi-Square | df^b^ | Sig. |
| PROBIT | Pearson Goodness-of-Fit Test | 3.659 | 2 | .161^a^ |
| a. Since the significance level is greater than .150, no heterogeneity factor is used in the calculation of confidence limits. | | | | |
| b. Statistics based on individual cases differ from statistics based on aggregated cases. | | | | |

| **Cell Counts and Residuals** | | | | | | | |
| --- | --- | --- | --- | --- | --- | --- | --- |
|  | Number | VAR00001 | Number of Subjects | Observed Responses | Expected Responses | Residual | Probability |
| PROBIT | 1 | 2.512 | 100 | 80 | 77.738 | 2.262 | .777 |
|  | 2 | 2.813 | 100 | 90 | 93.533 | -3.533 | .935 |
|  | 3 | 3.114 | 100 | 100 | 98.840 | 1.160 | .988 |
|  | 4 | 3.415 | 100 | 100 | 99.875 | .125 | .999 |

| **Confidence Limits** | | | | | | | |
| --- | --- | --- | --- | --- | --- | --- | --- |
|  | Probability | 95% Confidence Limits for VAR00001 | | | 95% Confidence Limits for log(VAR00001)^a^ | | |
|  |  | Estimate | Lower Bound | Upper Bound | Estimate | Lower Bound | Upper Bound |
| PROBIT | .010 | 18.935 | 2.297 | 47.678 | 1.277 | .361 | 1.678 |
|  | .020 | 24.333 | 3.473 | 57.141 | 1.386 | .541 | 1.757 |
|  | .030 | 28.530 | 4.513 | 64.103 | 1.455 | .654 | 1.807 |
|  | .040 | 32.158 | 5.496 | 69.899 | 1.507 | .740 | 1.844 |
|  | .050 | 35.447 | 6.451 | 75.002 | 1.550 | .810 | 1.875 |
|  | .060 | 38.510 | 7.394 | 79.641 | 1.586 | .869 | 1.901 |
|  | .070 | 41.413 | 8.333 | 83.946 | 1.617 | .921 | 1.924 |
|  | .080 | 44.197 | 9.274 | 88.001 | 1.645 | .967 | 1.944 |
|  | .090 | 46.892 | 10.221 | 91.860 | 1.671 | 1.009 | 1.963 |
|  | .100 | 49.517 | 11.178 | 95.565 | 1.695 | 1.048 | 1.980 |
|  | .150 | 62.044 | 16.190 | 112.590 | 1.793 | 1.209 | 2.051 |
|  | .200 | 74.224 | 21.723 | 128.307 | 1.871 | 1.337 | 2.108 |
|  | .250 | 86.562 | 27.946 | 143.579 | 1.937 | 1.446 | 2.157 |
|  | .300 | 99.380 | 35.027 | 158.893 | 1.997 | 1.544 | 2.201 |
|  | .350 | 112.947 | 43.164 | 174.605 | 2.053 | 1.635 | 2.242 |
|  | .400 | 127.529 | 52.605 | 191.029 | 2.106 | 1.721 | 2.281 |
|  | .450 | 143.427 | 63.668 | 208.495 | 2.157 | 1.804 | 2.319 |
|  | .500 | 161.007 | 76.776 | 227.384 | 2.207 | 1.885 | 2.357 |
|  | .550 | 180.741 | 92.508 | 248.186 | 2.257 | 1.966 | 2.395 |
|  | .600 | 203.272 | 111.675 | 271.574 | 2.308 | 2.048 | 2.434 |
|  | .650 | 229.516 | 135.448 | 298.549 | 2.361 | 2.132 | 2.475 |
|  | .700 | 260.847 | 165.578 | 330.721 | 2.416 | 2.219 | 2.519 |
|  | .750 | 299.475 | 204.751 | 370.967 | 2.476 | 2.311 | 2.569 |
|  | .800 | 349.256 | 257.163 | 425.191 | 2.543 | 2.410 | 2.629 |
|  | .850 | 417.819 | 329.190 | 507.908 | 2.621 | 2.517 | 2.706 |
|  | .900 | 523.520 | 430.413 | 662.847 | 2.719 | 2.634 | 2.821 |
|  | .910 | 552.829 | 455.730 | 712.266 | 2.743 | 2.659 | 2.853 |
|  | .920 | 586.530 | 483.522 | 772.370 | 2.768 | 2.684 | 2.888 |
|  | .930 | 625.963 | 514.486 | 846.879 | 2.797 | 2.711 | 2.928 |
|  | .940 | 673.146 | 549.714 | 941.533 | 2.828 | 2.740 | 2.974 |
|  | .950 | 731.314 | 590.970 | 1065.836 | 2.864 | 2.772 | 3.028 |
|  | .960 | 806.108 | 641.314 | 1237.035 | 2.906 | 2.807 | 3.092 |
|  | .970 | 908.620 | 706.653 | 1490.824 | 2.958 | 2.849 | 3.173 |
|  | .980 | 1065.351 | 800.706 | 1918.252 | 3.027 | 2.903 | 3.283 |
|  | .990 | 1369.051 | 969.516 | 2870.168 | 3.136 | 2.987 | 3.458 |
| a. Logarithm base = 10. | | | | | | | |


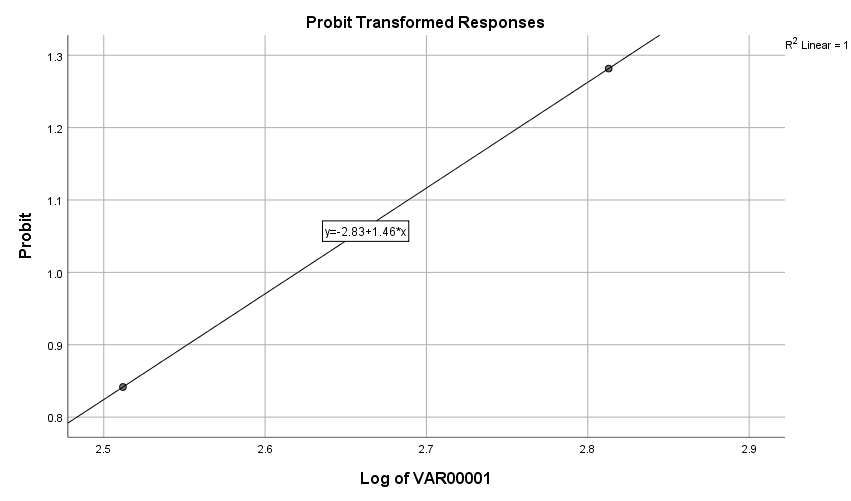


**Probit Analysis 17/7**

|  |
| --- |

| **Data Information** | | |
| --- | --- | --- |
|  | | N of Cases |
| Valid | | 4 |
| Rejected | Missing | 0 |
|  | LOG Transform Cannot be Done | 0 |
|  | Number of Responses > Number of Subjects | 0 |
| Control Group | | 1 |

| **Convergence Information** | | |
| --- | --- | --- |
|  | Number of Iterations | Optimal Solution Found |
| PROBIT | 20 | No^a^ |
| a. Parameter estimates did not converge. | | |

| **Parameter Estimates** | | | | | | | |
| --- | --- | --- | --- | --- | --- | --- | --- |
|  | Parameter | Estimate | Std. Error | Z | Sig. | 95% Confidence Interval | |
|  |  |  |  |  |  | Lower Bound | Upper Bound |
| PROBIT^a^ | VAR00001 | 3.320 | .583 | 5.695 | .000 | 2.178 | 4.463 |
|  | Intercept | -7.796 | 1.540 | -5.064 | .000 | -9.336 | -6.256 |
| a. PROBIT model: PROBIT(p) = Intercept + BX (Covariates X are transformed using the base 10.000 logarithm.) | | | | | | | |

| **Chi-Square Tests** | | | | |
| --- | --- | --- | --- | --- |
|  | | Chi-Square | df^b^ | Sig. |
| PROBIT | Pearson Goodness-of-Fit Test | .638 | 2 | .727^a^ |
| a. Since the significance level is greater than .150, no heterogeneity factor is used in the calculation of confidence limits. | | | | |
| b. Statistics based on individual cases differ from statistics based on aggregated cases. | | | | |

| **Cell Counts and Residuals** | | | | | | | |
| --- | --- | --- | --- | --- | --- | --- | --- |
|  | Number | VAR00001 | Number of Subjects | Observed Responses | Expected Responses | Residual | Probability |
| PROBIT | 1 | 2.512 | 100 | 70 | 70.693 | -.693 | .707 |
|  | 2 | 2.813 | 100 | 95 | 93.870 | 1.130 | .939 |
|  | 3 | 3.114 | 100 | 99 | 99.451 | -.451 | .995 |
|  | 4 | 3.415 | 100 | 100 | 99.980 | .020 | 1.000 |

| **Confidence Limits** | | | | | | | |
| --- | --- | --- | --- | --- | --- | --- | --- |
|  | Probability | 95% Confidence Limits for VAR00001 | | | 95% Confidence Limits for log(VAR00001)^a^ | | |
|  |  | Estimate | Lower Bound | Upper Bound | Estimate | Lower Bound | Upper Bound |
| PROBIT | .010 | 44.391 | 13.292 | 80.499 | 1.647 | 1.124 | 1.906 |
|  | .020 | 53.628 | 17.712 | 92.766 | 1.729 | 1.248 | 1.967 |
|  | .030 | 60.461 | 21.247 | 101.512 | 1.781 | 1.327 | 2.007 |
|  | .040 | 66.170 | 24.363 | 108.640 | 1.821 | 1.387 | 2.036 |
|  | .050 | 71.209 | 27.230 | 114.811 | 1.853 | 1.435 | 2.060 |
|  | .060 | 75.799 | 29.934 | 120.343 | 1.880 | 1.476 | 2.080 |
|  | .070 | 80.067 | 32.523 | 125.418 | 1.903 | 1.512 | 2.098 |
|  | .080 | 84.091 | 35.030 | 130.147 | 1.925 | 1.544 | 2.114 |
|  | .090 | 87.927 | 37.476 | 134.605 | 1.944 | 1.574 | 2.129 |
|  | .100 | 91.612 | 39.878 | 138.848 | 1.962 | 1.601 | 2.143 |
|  | .150 | 108.586 | 51.557 | 157.932 | 2.036 | 1.712 | 2.198 |
|  | .200 | 124.292 | 63.207 | 175.027 | 2.094 | 1.801 | 2.243 |
|  | .250 | 139.566 | 75.248 | 191.237 | 2.145 | 1.876 | 2.282 |
|  | .300 | 154.875 | 87.967 | 207.155 | 2.190 | 1.944 | 2.316 |
|  | .350 | 170.556 | 101.620 | 223.187 | 2.232 | 2.007 | 2.349 |
|  | .400 | 186.902 | 116.469 | 239.669 | 2.272 | 2.066 | 2.380 |
|  | .450 | 204.206 | 132.814 | 256.934 | 2.310 | 2.123 | 2.410 |
|  | .500 | 222.800 | 151.020 | 275.355 | 2.348 | 2.179 | 2.440 |
|  | .550 | 243.086 | 171.544 | 295.403 | 2.386 | 2.234 | 2.470 |
|  | .600 | 265.592 | 194.977 | 317.724 | 2.424 | 2.290 | 2.502 |
|  | .650 | 291.046 | 222.099 | 343.291 | 2.464 | 2.347 | 2.536 |
|  | .700 | 320.515 | 253.942 | 373.683 | 2.506 | 2.405 | 2.573 |
|  | .750 | 355.672 | 291.869 | 411.721 | 2.551 | 2.465 | 2.615 |
|  | .800 | 399.379 | 337.664 | 462.918 | 2.601 | 2.528 | 2.666 |
|  | .850 | 457.147 | 393.982 | 539.042 | 2.660 | 2.595 | 2.732 |
|  | .900 | 541.849 | 467.209 | 668.454 | 2.734 | 2.670 | 2.825 |
|  | .910 | 564.558 | 485.318 | 706.332 | 2.752 | 2.686 | 2.849 |
|  | .920 | 590.308 | 505.254 | 750.709 | 2.771 | 2.704 | 2.875 |
|  | .930 | 619.979 | 527.550 | 803.597 | 2.792 | 2.722 | 2.905 |
|  | .940 | 654.883 | 553.002 | 868.046 | 2.816 | 2.743 | 2.939 |
|  | .950 | 697.097 | 582.853 | 948.986 | 2.843 | 2.766 | 2.977 |
|  | .960 | 750.182 | 619.214 | 1055.094 | 2.875 | 2.792 | 3.023 |
|  | .970 | 821.015 | 666.097 | 1203.633 | 2.914 | 2.824 | 3.080 |
|  | .980 | 925.635 | 732.680 | 1436.460 | 2.966 | 2.865 | 3.157 |
|  | .990 | 1118.248 | 849.120 | 1903.267 | 3.049 | 2.929 | 3.279 |
| a. Logarithm base = 10. | | | | | | | |


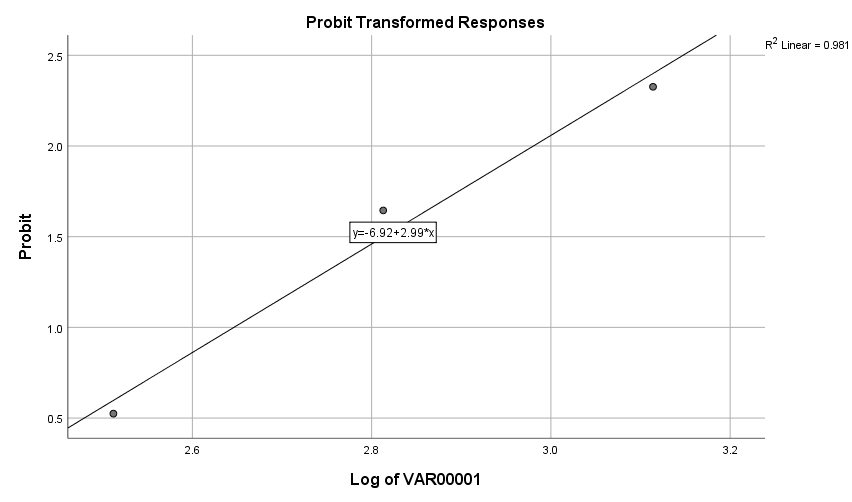


**Probit Analysis 17/12**

| **Data Information** | | |
| --- | --- | --- |
|  | | N of Cases |
| Valid | | 4 |
| Rejected | Missing | 0 |
|  | LOG Transform Cannot be Done | 0 |
|  | Number of Responses > Number of Subjects | 0 |
| Control Group | | 1 |

| **Convergence Information** | | |
| --- | --- | --- |
|  | Number of Iterations | Optimal Solution Found |
| PROBIT | 13 | Yes |

| **Parameter Estimates** | | | | | | | |
| --- | --- | --- | --- | --- | --- | --- | --- |
|  | Parameter | Estimate | Std. Error | Z | Sig. | 95% Confidence Interval | |
|  |  |  |  |  |  | Lower Bound | Upper Bound |
| PROBIT^a^ | VAR00001 | 2.420 | .368 | 6.568 | .000 | 1.698 | 3.142 |
|  | Intercept | -5.663 | 1.008 | -5.621 | .000 | -6.670 | -4.655 |
| a. PROBIT model: PROBIT(p) = Intercept + BX (Covariates X are transformed using the base 10.000 logarithm.) | | | | | | | |

| **Chi-Square Tests** | | | | |
| --- | --- | --- | --- | --- |
|  | | Chi-Square | df^b^ | Sig. |
| PROBIT | Pearson Goodness-of-Fit Test | 2.421 | 2 | .298^a^ |
| a. Since the significance level is greater than .150, no heterogeneity factor is used in the calculation of confidence limits. | | | | |
| b. Statistics based on individual cases differ from statistics based on aggregated cases. | | | | |

| **Cell Counts and Residuals** | | | | | | | |
| --- | --- | --- | --- | --- | --- | --- | --- |
|  | Number | VAR00001 | Number of Subjects | Observed Responses | Expected Responses | Residual | Probability |
| PROBIT | 1 | 2.512 | 100 | 65 | 66.101 | -1.101 | .661 |
|  | 2 | 2.813 | 100 | 90 | 87.361 | 2.639 | .874 |
|  | 3 | 3.114 | 100 | 95 | 96.940 | -1.940 | .969 |
|  | 4 | 3.415 | 100 | 100 | 99.535 | .465 | .995 |

| **Confidence Limits** | | | | | | | |
| --- | --- | --- | --- | --- | --- | --- | --- |
|  | Probability | 95% Confidence Limits for VAR00001 | | | 95% Confidence Limits for log(VAR00001)^a^ | | |
|  |  | Estimate | Lower Bound | Upper Bound | Estimate | Lower Bound | Upper Bound |
| PROBIT | .010 | 23.927 | 6.312 | 49.516 | 1.379 | .800 | 1.695 |
|  | .020 | 31.013 | 9.123 | 60.551 | 1.492 | .960 | 1.782 |
|  | .030 | 36.562 | 11.523 | 68.805 | 1.563 | 1.062 | 1.838 |
|  | .040 | 41.380 | 13.735 | 75.754 | 1.617 | 1.138 | 1.879 |
|  | .050 | 45.765 | 15.843 | 81.927 | 1.661 | 1.200 | 1.913 |
|  | .060 | 49.861 | 17.889 | 87.579 | 1.698 | 1.253 | 1.942 |
|  | .070 | 53.753 | 19.899 | 92.859 | 1.730 | 1.299 | 1.968 |
|  | .080 | 57.494 | 21.888 | 97.860 | 1.760 | 1.340 | 1.991 |
|  | .090 | 61.123 | 23.869 | 102.644 | 1.786 | 1.378 | 2.011 |
|  | .100 | 64.665 | 25.850 | 107.258 | 1.811 | 1.412 | 2.030 |
|  | .150 | 81.653 | 35.945 | 128.720 | 1.912 | 1.556 | 2.110 |
|  | .200 | 98.283 | 46.690 | 148.876 | 1.992 | 1.669 | 2.173 |
|  | .250 | 115.226 | 58.406 | 168.747 | 2.062 | 1.766 | 2.227 |
|  | .300 | 132.916 | 71.377 | 188.936 | 2.124 | 1.854 | 2.276 |
|  | .350 | 151.724 | 85.908 | 209.908 | 2.181 | 1.934 | 2.322 |
|  | .400 | 172.026 | 102.358 | 232.103 | 2.236 | 2.010 | 2.366 |
|  | .450 | 194.250 | 121.174 | 256.003 | 2.288 | 2.083 | 2.408 |
|  | .500 | 218.924 | 142.930 | 282.195 | 2.340 | 2.155 | 2.451 |
|  | .550 | 246.731 | 168.378 | 311.460 | 2.392 | 2.226 | 2.493 |
|  | .600 | 278.607 | 198.532 | 344.912 | 2.445 | 2.298 | 2.538 |
|  | .650 | 315.887 | 234.779 | 384.257 | 2.500 | 2.371 | 2.585 |
|  | .700 | 360.586 | 279.044 | 432.314 | 2.557 | 2.446 | 2.636 |
|  | .750 | 415.945 | 334.021 | 494.171 | 2.619 | 2.524 | 2.694 |
|  | .800 | 487.647 | 403.574 | 579.924 | 2.688 | 2.606 | 2.763 |
|  | .850 | 586.968 | 494.047 | 711.678 | 2.769 | 2.694 | 2.852 |
|  | .900 | 741.162 | 620.543 | 945.545 | 2.870 | 2.793 | 2.976 |
|  | .910 | 784.115 | 653.406 | 1016.218 | 2.894 | 2.815 | 3.007 |
|  | .920 | 833.604 | 690.290 | 1100.259 | 2.921 | 2.839 | 3.041 |
|  | .930 | 891.632 | 732.393 | 1202.119 | 2.950 | 2.865 | 3.080 |
|  | .940 | 961.228 | 781.513 | 1328.674 | 2.983 | 2.893 | 3.123 |
|  | .950 | 1047.257 | 840.503 | 1491.238 | 3.020 | 2.925 | 3.174 |
|  | .960 | 1158.217 | 914.275 | 1710.150 | 3.064 | 2.961 | 3.233 |
|  | .970 | 1310.868 | 1012.335 | 2026.919 | 3.118 | 3.005 | 3.307 |
|  | .980 | 1545.380 | 1156.940 | 2545.518 | 3.189 | 3.063 | 3.406 |
|  | .990 | 2003.047 | 1423.715 | 3655.944 | 3.302 | 3.153 | 3.563 |
| a. Logarithm base = 10. | | | | | | | |


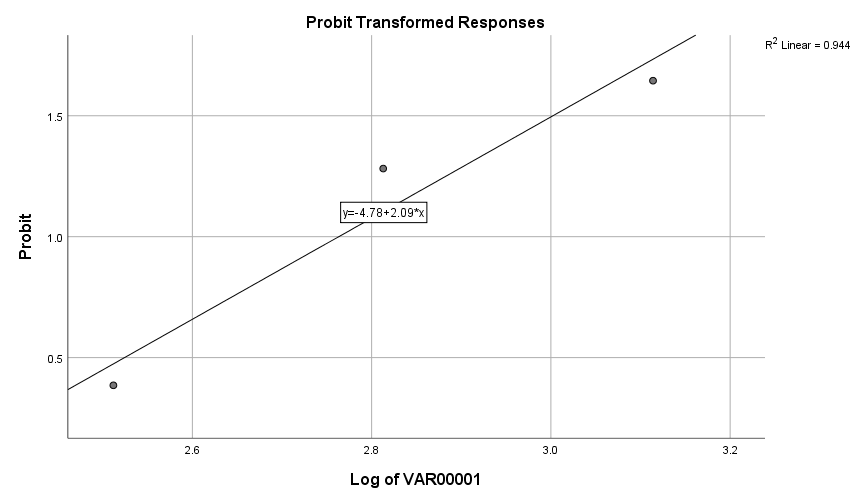


**Probit Analysis 17/13**

| **Data Information** | | |
| --- | --- | --- |
|  | | N of Cases |
| Valid | | 4 |
| Rejected | Missing | 0 |
|  | LOG Transform Cannot be Done | 0 |
|  | Number of Responses > Number of Subjects | 0 |
| Control Group | | 1 |

| **Convergence Information** | | |
| --- | --- | --- |
|  | Number of Iterations | Optimal Solution Found |
| PROBIT | 14 | Yes |

| **Parameter Estimates** | | | | | | | |
| --- | --- | --- | --- | --- | --- | --- | --- |
|  | Parameter | Estimate | Std. Error | Z | Sig. | 95% Confidence Interval | |
|  |  |  |  |  |  | Lower Bound | Upper Bound |
| PROBIT^a^ | VAR00001 | 2.571 | .287 | 8.961 | .000 | 2.009 | 3.134 |
|  | Intercept | -6.612 | .805 | -8.213 | .000 | -7.417 | -5.807 |
| a. PROBIT model: PROBIT(p) = Intercept + BX (Covariates X are transformed using the base 10.000 logarithm.) | | | | | | | |

| **Chi-Square Tests** | | | | |
| --- | --- | --- | --- | --- |
|  | | Chi-Square | df^b^ | Sig. |
| PROBIT | Pearson Goodness-of-Fit Test | 3.560 | 2 | .169^a^ |
| a. Since the significance level is greater than .150, no heterogeneity factor is used in the calculation of confidence limits. | | | | |
| b. Statistics based on individual cases differ from statistics based on aggregated cases. | | | | |

| **Cell Counts and Residuals** | | | | | | | |
| --- | --- | --- | --- | --- | --- | --- | --- |
|  | Number | VAR00001 | Number of Subjects | Observed Responses | Expected Responses | Residual | Probability |
| PROBIT | 1 | 2.512 | 100 | 40 | 43.925 | -3.925 | .439 |
|  | 2 | 2.813 | 100 | 80 | 73.277 | 6.723 | .733 |
|  | 3 | 3.114 | 100 | 90 | 91.854 | -1.854 | .919 |
|  | 4 | 3.415 | 100 | 98 | 98.497 | -.497 | .985 |

| **Confidence Limits** | | | | | | | |
| --- | --- | --- | --- | --- | --- | --- | --- |
|  | Probability | 95% Confidence Limits for VAR00001 | | | 95% Confidence Limits for log(VAR00001)^a^ | | |
|  |  | Estimate | Lower Bound | Upper Bound | Estimate | Lower Bound | Upper Bound |
| PROBIT | .010 | 46.415 | 22.097 | 75.150 | 1.667 | 1.344 | 1.876 |
|  | .020 | 59.247 | 30.149 | 91.972 | 1.773 | 1.479 | 1.964 |
|  | .030 | 69.171 | 36.713 | 104.567 | 1.840 | 1.565 | 2.019 |
|  | .040 | 77.718 | 42.571 | 115.180 | 1.891 | 1.629 | 2.061 |
|  | .050 | 85.443 | 48.015 | 124.613 | 1.932 | 1.681 | 2.096 |
|  | .060 | 92.621 | 53.189 | 133.258 | 1.967 | 1.726 | 2.125 |
|  | .070 | 99.408 | 58.180 | 141.338 | 1.997 | 1.765 | 2.150 |
|  | .080 | 105.907 | 63.042 | 148.996 | 2.025 | 1.800 | 2.173 |
|  | .090 | 112.185 | 67.812 | 156.327 | 2.050 | 1.831 | 2.194 |
|  | .100 | 118.293 | 72.518 | 163.401 | 2.073 | 1.860 | 2.213 |
|  | .150 | 147.326 | 95.688 | 196.365 | 2.168 | 1.981 | 2.293 |
|  | .200 | 175.404 | 119.183 | 227.425 | 2.244 | 2.076 | 2.357 |
|  | .250 | 203.720 | 143.776 | 258.158 | 2.309 | 2.158 | 2.412 |
|  | .300 | 233.024 | 170.018 | 289.519 | 2.367 | 2.230 | 2.462 |
|  | .350 | 263.929 | 198.409 | 322.268 | 2.421 | 2.298 | 2.508 |
|  | .400 | 297.036 | 229.470 | 357.152 | 2.473 | 2.361 | 2.553 |
|  | .450 | 333.014 | 263.791 | 395.020 | 2.522 | 2.421 | 2.597 |
|  | .500 | 372.675 | 302.067 | 436.940 | 2.571 | 2.480 | 2.640 |
|  | .550 | 417.059 | 345.140 | 484.367 | 2.620 | 2.538 | 2.685 |
|  | .600 | 467.576 | 394.061 | 539.394 | 2.670 | 2.596 | 2.732 |
|  | .650 | 526.228 | 450.190 | 605.168 | 2.721 | 2.653 | 2.782 |
|  | .700 | 596.019 | 515.414 | 686.628 | 2.775 | 2.712 | 2.837 |
|  | .750 | 681.753 | 592.685 | 791.869 | 2.834 | 2.773 | 2.899 |
|  | .800 | 791.810 | 687.297 | 935.088 | 2.899 | 2.837 | 2.971 |
|  | .850 | 942.714 | 810.110 | 1144.418 | 2.974 | 2.909 | 3.059 |
|  | .900 | 1174.092 | 987.491 | 1488.739 | 3.070 | 2.995 | 3.173 |
|  | .910 | 1238.013 | 1034.783 | 1588.042 | 3.093 | 3.015 | 3.201 |
|  | .920 | 1311.404 | 1088.350 | 1704.039 | 3.118 | 3.037 | 3.231 |
|  | .930 | 1397.134 | 1150.033 | 1842.062 | 3.145 | 3.061 | 3.265 |
|  | .940 | 1499.522 | 1222.588 | 2010.254 | 3.176 | 3.087 | 3.303 |
|  | .950 | 1625.487 | 1310.380 | 2221.852 | 3.211 | 3.117 | 3.347 |
|  | .960 | 1787.062 | 1420.923 | 2500.291 | 3.252 | 3.153 | 3.398 |
|  | .970 | 2007.872 | 1568.764 | 2892.549 | 3.303 | 3.196 | 3.461 |
|  | .980 | 2344.203 | 1787.982 | 3513.603 | 3.370 | 3.252 | 3.546 |
|  | .990 | 2992.291 | 2194.450 | 4780.361 | 3.476 | 3.341 | 3.679 |
| a. Logarithm base = 10. | | | | | | | |


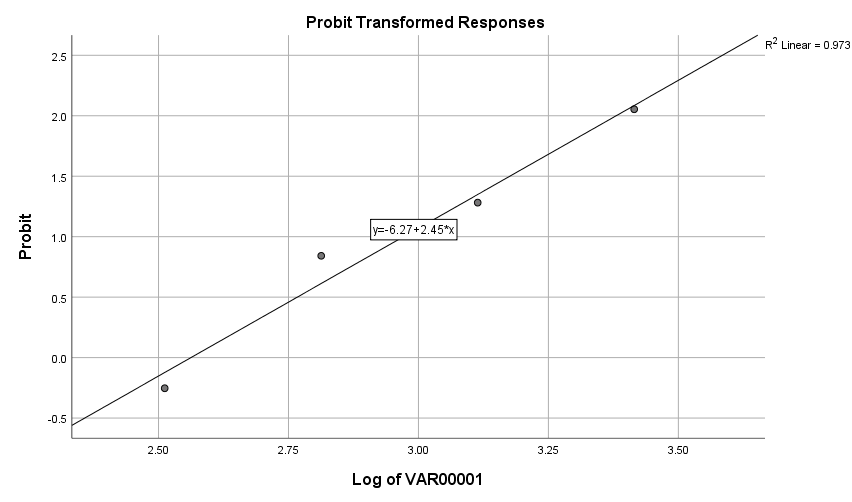


**Probit Analysis 17/16**

| **Data Information** | | |
| --- | --- | --- |
|  | | N of Cases |
| Valid | | 4 |
| Rejected | Missing | 0 |
|  | LOG Transform Cannot be Done | 0 |
|  | Number of Responses > Number of Subjects | 0 |
| Control Group | | 1 |

| **Convergence Information** | | |
| --- | --- | --- |
|  | Number of Iterations | Optimal Solution Found |
| PROBIT | 10 | Yes |

| **Parameter Estimates** | | | | | | | |
| --- | --- | --- | --- | --- | --- | --- | --- |
|  | Parameter | Estimate | Std. Error | Z | Sig. | 95% Confidence Interval | |
|  |  |  |  |  |  | Lower Bound | Upper Bound |
| PROBIT^a^ | VAR00001 | 1.477 | .201 | 7.337 | .000 | 1.082 | 1.871 |
|  | Intercept | -4.483 | .603 | -7.437 | .000 | -5.086 | -3.880 |
| a. PROBIT model: PROBIT(p) = Intercept + BX (Covariates X are transformed using the base 10.000 logarithm.) | | | | | | | |

| **Chi-Square Tests** | | | | |
| --- | --- | --- | --- | --- |
|  | | Chi-Square | df^b^ | Sig. |
| PROBIT | Pearson Goodness-of-Fit Test | .663 | 2 | .718^a^ |
| a. Since the significance level is greater than .150, no heterogeneity factor is used in the calculation of confidence limits. | | | | |
| b. Statistics based on individual cases differ from statistics based on aggregated cases. | | | | |

| **Cell Counts and Residuals** | | | | | | | |
| --- | --- | --- | --- | --- | --- | --- | --- |
|  | Number | VAR00001 | Number of Subjects | Observed Responses | Expected Responses | Residual | Probability |
| PROBIT | 1 | 2.512 | 100 | 20 | 21.949 | -1.949 | .219 |
|  | 2 | 2.813 | 100 | 40 | 37.092 | 2.908 | .371 |
|  | 3 | 3.114 | 100 | 55 | 54.581 | .419 | .546 |
|  | 4 | 3.415 | 100 | 70 | 71.212 | -1.212 | .712 |

| **Confidence Limits** | | | | | | | |
| --- | --- | --- | --- | --- | --- | --- | --- |
|  | Probability | 95% Confidence Limits for VAR00001 | | | 95% Confidence Limits for log(VAR00001)^a^ | | |
|  |  | Estimate | Lower Bound | Upper Bound | Estimate | Lower Bound | Upper Bound |
| PROBIT | .010 | 28.877 | 7.914 | 61.603 | 1.461 | .898 | 1.790 |
|  | .020 | 44.174 | 14.094 | 86.406 | 1.645 | 1.149 | 1.937 |
|  | .030 | 57.849 | 20.318 | 107.143 | 1.762 | 1.308 | 2.030 |
|  | .040 | 70.861 | 26.744 | 125.999 | 1.850 | 1.427 | 2.100 |
|  | .050 | 83.575 | 33.437 | 143.792 | 1.922 | 1.524 | 2.158 |
|  | .060 | 96.179 | 40.429 | 160.935 | 1.983 | 1.607 | 2.207 |
|  | .070 | 108.785 | 47.745 | 177.670 | 2.037 | 1.679 | 2.250 |
|  | .080 | 121.468 | 55.402 | 194.158 | 2.084 | 1.744 | 2.288 |
|  | .090 | 134.282 | 63.419 | 210.510 | 2.128 | 1.802 | 2.323 |
|  | .100 | 147.270 | 71.809 | 226.810 | 2.168 | 1.856 | 2.356 |
|  | .150 | 215.830 | 119.873 | 309.476 | 2.334 | 2.079 | 2.491 |
|  | .200 | 292.445 | 179.510 | 397.555 | 2.466 | 2.254 | 2.599 |
|  | .250 | 379.515 | 252.765 | 494.920 | 2.579 | 2.403 | 2.695 |
|  | .300 | 479.593 | 341.823 | 605.839 | 2.681 | 2.534 | 2.782 |
|  | .350 | 595.746 | 448.755 | 736.209 | 2.775 | 2.652 | 2.867 |
|  | .400 | 731.874 | 575.064 | 894.910 | 2.864 | 2.760 | 2.952 |
|  | .450 | 893.116 | 721.414 | 1095.324 | 2.951 | 2.858 | 3.040 |
|  | .500 | 1086.447 | 888.371 | 1356.477 | 3.036 | 2.949 | 3.132 |
|  | .550 | 1321.628 | 1078.434 | 1704.091 | 3.121 | 3.033 | 3.231 |
|  | .600 | 1612.801 | 1297.954 | 2173.996 | 3.208 | 3.113 | 3.337 |
|  | .650 | 1981.327 | 1558.228 | 2820.803 | 3.297 | 3.193 | 3.450 |
|  | .700 | 2461.185 | 1877.265 | 3735.331 | 3.391 | 3.274 | 3.572 |
|  | .750 | 3110.197 | 2284.503 | 5081.254 | 3.493 | 3.359 | 3.706 |
|  | .800 | 4036.204 | 2832.452 | 7183.969 | 3.606 | 3.452 | 3.856 |
|  | .850 | 5468.954 | 3628.073 | 10789.171 | 3.738 | 3.560 | 4.033 |
|  | .900 | 8015.004 | 4939.772 | 18049.557 | 3.904 | 3.694 | 4.256 |
|  | .910 | 8790.178 | 5320.297 | 20444.992 | 3.944 | 3.726 | 4.311 |
|  | .920 | 9717.493 | 5766.324 | 23411.571 | 3.988 | 3.761 | 4.369 |
|  | .930 | 10850.432 | 6299.275 | 27175.947 | 4.035 | 3.799 | 4.434 |
|  | .940 | 12272.577 | 6952.017 | 32104.210 | 4.089 | 3.842 | 4.507 |
|  | .950 | 14123.424 | 7778.277 | 38830.479 | 4.150 | 3.891 | 4.589 |
|  | .960 | 16657.586 | 8873.806 | 48562.920 | 4.222 | 3.948 | 4.686 |
|  | .970 | 20404.398 | 10432.033 | 63945.506 | 4.310 | 4.018 | 4.806 |
|  | .980 | 26721.017 | 12931.087 | 92215.182 | 4.427 | 4.112 | 4.965 |
|  | .990 | 40875.499 | 18130.084 | 164296.483 | 4.611 | 4.258 | 5.216 |
| a. Logarithm base = 10. | | | | | | | |


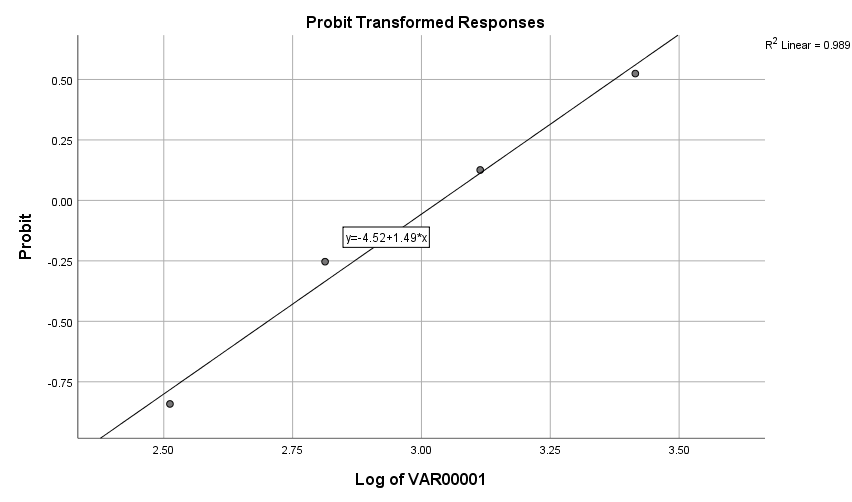


**Probit Analysis 17/17**

| **Data Information** | | |
| --- | --- | --- |
|  | | N of Cases |
| Valid | | 4 |
| Rejected | Missing | 0 |
|  | LOG Transform Cannot be Done | 0 |
|  | Number of Responses > Number of Subjects | 0 |
| Control Group | | 1 |

| **Convergence Information** | | |
| --- | --- | --- |
|  | Number of Iterations | Optimal Solution Found |
| PROBIT | 20 | No^a^ |
| a. Parameter estimates did not converge. | | |

| **Parameter Estimates** | | | | | | | |
| --- | --- | --- | --- | --- | --- | --- | --- |
|  | Parameter | Estimate | Std. Error | Z | Sig. | 95% Confidence Interval | |
|  |  |  |  |  |  | Lower Bound | Upper Bound |
| PROBIT^a^ | VAR00001 | 3.179 | .543 | 5.859 | .000 | 2.116 | 4.243 |
|  | Intercept | -7.506 | 1.441 | -5.209 | .000 | -8.947 | -6.065 |
| a. PROBIT model: PROBIT(p) = Intercept + BX (Covariates X are transformed using the base 10.000 logarithm.) | | | | | | | |

| **Chi-Square Tests** | | | | |
| --- | --- | --- | --- | --- |
|  | | Chi-Square | df^b^ | Sig. |
| PROBIT | Pearson Goodness-of-Fit Test | 1.864 | 2 | .394^a^ |
| a. Since the significance level is greater than .150, no heterogeneity factor is used in the calculation of confidence limits. | | | | |
| b. Statistics based on individual cases differ from statistics based on aggregated cases. | | | | |

| **Cell Counts and Residuals** | | | | | | | |
| --- | --- | --- | --- | --- | --- | --- | --- |
|  | Number | VAR00001 | Number of Subjects | Observed Responses | Expected Responses | Residual | Probability |
| PROBIT | 1 | 2.512 | 100 | 70 | 68.431 | 1.569 | .684 |
|  | 2 | 2.813 | 100 | 90 | 92.462 | -2.462 | .925 |
|  | 3 | 3.114 | 100 | 100 | 99.166 | .834 | .992 |
|  | 4 | 3.415 | 100 | 100 | 99.960 | .040 | 1.000 |

| **Confidence Limits** | | | | | | | |
| --- | --- | --- | --- | --- | --- | --- | --- |
|  | Probability | 95% Confidence Limits for VAR00001 | | | 95% Confidence Limits for log(VAR00001)^a^ | | |
|  |  | Estimate | Lower Bound | Upper Bound | Estimate | Lower Bound | Upper Bound |
| PROBIT | .010 | 42.582 | 12.909 | 77.733 | 1.629 | 1.111 | 1.891 |
|  | .020 | 51.876 | 17.347 | 90.237 | 1.715 | 1.239 | 1.955 |
|  | .030 | 58.799 | 20.921 | 99.205 | 1.769 | 1.321 | 1.997 |
|  | .040 | 64.609 | 24.086 | 106.542 | 1.810 | 1.382 | 2.028 |
|  | .050 | 69.756 | 27.009 | 112.915 | 1.844 | 1.432 | 2.053 |
|  | .060 | 74.459 | 29.773 | 118.644 | 1.872 | 1.474 | 2.074 |
|  | .070 | 78.843 | 32.427 | 123.910 | 1.897 | 1.511 | 2.093 |
|  | .080 | 82.986 | 35.003 | 128.827 | 1.919 | 1.544 | 2.110 |
|  | .090 | 86.944 | 37.522 | 133.472 | 1.939 | 1.574 | 2.125 |
|  | .100 | 90.753 | 39.999 | 137.899 | 1.958 | 1.602 | 2.140 |
|  | .150 | 108.383 | 52.105 | 157.895 | 2.035 | 1.717 | 2.198 |
|  | .200 | 124.807 | 64.261 | 175.913 | 2.096 | 1.808 | 2.245 |
|  | .250 | 140.867 | 76.895 | 193.082 | 2.149 | 1.886 | 2.286 |
|  | .300 | 157.043 | 90.305 | 210.015 | 2.196 | 1.956 | 2.322 |
|  | .350 | 173.686 | 104.759 | 227.137 | 2.240 | 2.020 | 2.356 |
|  | .400 | 191.107 | 120.543 | 244.809 | 2.281 | 2.081 | 2.389 |
|  | .450 | 209.624 | 137.981 | 263.390 | 2.321 | 2.140 | 2.421 |
|  | .500 | 229.597 | 157.471 | 283.292 | 2.361 | 2.197 | 2.452 |
|  | .550 | 251.474 | 179.511 | 305.041 | 2.400 | 2.254 | 2.484 |
|  | .600 | 275.839 | 204.748 | 329.367 | 2.441 | 2.311 | 2.518 |
|  | .650 | 303.507 | 234.026 | 357.374 | 2.482 | 2.369 | 2.553 |
|  | .700 | 335.672 | 268.454 | 390.877 | 2.526 | 2.429 | 2.592 |
|  | .750 | 374.218 | 309.469 | 433.125 | 2.573 | 2.491 | 2.637 |
|  | .800 | 422.373 | 358.942 | 490.453 | 2.626 | 2.555 | 2.691 |
|  | .850 | 486.377 | 419.796 | 576.216 | 2.687 | 2.623 | 2.761 |
|  | .900 | 580.864 | 499.511 | 722.295 | 2.764 | 2.699 | 2.859 |
|  | .910 | 606.312 | 519.394 | 765.071 | 2.783 | 2.715 | 2.884 |
|  | .920 | 635.224 | 541.364 | 815.210 | 2.803 | 2.733 | 2.911 |
|  | .930 | 668.608 | 566.035 | 875.007 | 2.825 | 2.753 | 2.942 |
|  | .940 | 707.971 | 594.316 | 947.955 | 2.850 | 2.774 | 2.977 |
|  | .950 | 755.700 | 627.631 | 1039.708 | 2.878 | 2.798 | 3.017 |
|  | .960 | 815.903 | 668.403 | 1160.238 | 2.912 | 2.825 | 3.065 |
|  | .970 | 896.527 | 721.241 | 1329.445 | 2.953 | 2.858 | 3.124 |
|  | .980 | 1016.169 | 796.726 | 1595.733 | 3.007 | 2.901 | 3.203 |
|  | .990 | 1237.973 | 929.758 | 2133.059 | 3.093 | 2.968 | 3.329 |
| a. Logarithm base = 10. | | | | | | | |


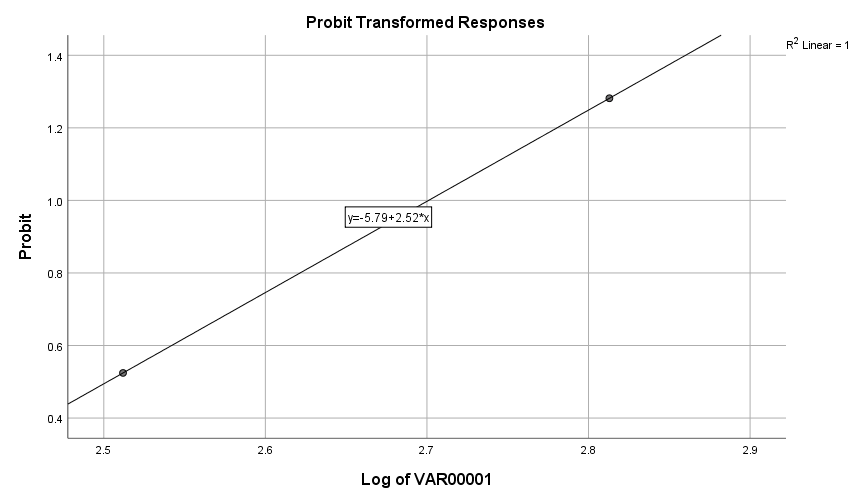


**Probit Analysis 17/25**

| **Data Information** | | |
| --- | --- | --- |
|  | | N of Cases |
| Valid | | 4 |
| Rejected | Missing | 0 |
|  | LOG Transform Cannot be Done | 0 |
|  | Number of Responses > Number of Subjects | 0 |
| Control Group | | 1 |

| **Convergence Information** | | |
| --- | --- | --- |
|  | Number of Iterations | Optimal Solution Found |
| PROBIT | 11 | Yes |

| **Parameter Estimates** | | | | | | | |
| --- | --- | --- | --- | --- | --- | --- | --- |
|  | Parameter | Estimate | Std. Error | Z | Sig. | 95% Confidence Interval | |
|  |  |  |  |  |  | Lower Bound | Upper Bound |
| PROBIT^a^ | VAR00001 | 1.361 | .227 | 5.983 | .000 | .915 | 1.806 |
|  | Intercept | -3.241 | .658 | -4.929 | .000 | -3.899 | -2.584 |
| a. PROBIT model: PROBIT(p) = Intercept + BX (Covariates X are transformed using the base 10.000 logarithm.) | | | | | | | |

| **Chi-Square Tests** | | | | |
| --- | --- | --- | --- | --- |
|  | | Chi-Square | df^b^ | Sig. |
| PROBIT | Pearson Goodness-of-Fit Test | 3.019 | 2 | .221^a^ |
| a. Since the significance level is greater than .150, no heterogeneity factor is used in the calculation of confidence limits. | | | | |
| b. Statistics based on individual cases differ from statistics based on aggregated cases. | | | | |

| **Cell Counts and Residuals** | | | | | | | |
| --- | --- | --- | --- | --- | --- | --- | --- |
|  | Number | VAR00001 | Number of Subjects | Observed Responses | Expected Responses | Residual | Probability |
| PROBIT | 1 | 2.512 | 100 | 60 | 57.011 | 2.989 | .570 |
|  | 2 | 2.813 | 100 | 70 | 72.115 | -2.115 | .721 |
|  | 3 | 3.114 | 100 | 80 | 84.034 | -4.034 | .840 |
|  | 4 | 3.415 | 100 | 95 | 92.006 | 2.994 | .920 |

| **Confidence Limits** | | | | | | | |
| --- | --- | --- | --- | --- | --- | --- | --- |
|  | Probability | 95% Confidence Limits for VAR00001 | | | 95% Confidence Limits for log(VAR00001)^a^ | | |
|  |  | Estimate | Lower Bound | Upper Bound | Estimate | Lower Bound | Upper Bound |
| PROBIT | .010 | 4.703 | .391 | 16.716 | .672 | -.408 | 1.223 |
|  | .020 | 7.460 | .775 | 23.702 | .873 | -.111 | 1.375 |
|  | .030 | 9.996 | 1.196 | 29.585 | 1.000 | .078 | 1.471 |
|  | .040 | 12.458 | 1.658 | 34.959 | 1.095 | .220 | 1.544 |
|  | .050 | 14.901 | 2.162 | 40.046 | 1.173 | .335 | 1.603 |
|  | .060 | 17.355 | 2.710 | 44.957 | 1.239 | .433 | 1.653 |
|  | .070 | 19.837 | 3.303 | 49.760 | 1.297 | .519 | 1.697 |
|  | .080 | 22.359 | 3.944 | 54.498 | 1.349 | .596 | 1.736 |
|  | .090 | 24.930 | 4.633 | 59.199 | 1.397 | .666 | 1.772 |
|  | .100 | 27.557 | 5.373 | 63.887 | 1.440 | .730 | 1.805 |
|  | .150 | 41.722 | 9.921 | 87.637 | 1.620 | .997 | 1.943 |
|  | .200 | 58.015 | 16.138 | 112.756 | 1.764 | 1.208 | 2.052 |
|  | .250 | 76.978 | 24.479 | 140.083 | 1.886 | 1.389 | 2.146 |
|  | .300 | 99.236 | 35.555 | 170.375 | 1.997 | 1.551 | 2.231 |
|  | .350 | 125.569 | 50.196 | 204.474 | 2.099 | 1.701 | 2.311 |
|  | .400 | 156.990 | 69.539 | 243.431 | 2.196 | 1.842 | 2.386 |
|  | .450 | 194.855 | 95.162 | 288.656 | 2.290 | 1.978 | 2.460 |
|  | .500 | 241.025 | 129.280 | 342.151 | 2.382 | 2.112 | 2.534 |
|  | .550 | 298.135 | 175.028 | 406.952 | 2.474 | 2.243 | 2.610 |
|  | .600 | 370.042 | 236.842 | 488.013 | 2.568 | 2.374 | 2.688 |
|  | .650 | 462.637 | 320.813 | 594.204 | 2.665 | 2.506 | 2.774 |
|  | .700 | 585.401 | 434.648 | 743.101 | 2.767 | 2.638 | 2.871 |
|  | .750 | 754.669 | 586.828 | 972.299 | 2.878 | 2.769 | 2.988 |
|  | .800 | 1001.347 | 788.386 | 1363.821 | 3.001 | 2.897 | 3.135 |
|  | .850 | 1392.374 | 1067.885 | 2107.339 | 3.144 | 3.029 | 3.324 |
|  | .900 | 2108.133 | 1513.138 | 3766.805 | 3.324 | 3.180 | 3.576 |
|  | .910 | 2330.278 | 1641.109 | 4347.046 | 3.367 | 3.215 | 3.638 |
|  | .920 | 2598.215 | 1790.915 | 5083.412 | 3.415 | 3.253 | 3.706 |
|  | .930 | 2928.520 | 1969.840 | 6042.832 | 3.467 | 3.294 | 3.781 |
|  | .940 | 3347.295 | 2189.081 | 7335.948 | 3.525 | 3.340 | 3.865 |
|  | .950 | 3898.482 | 2466.983 | 9159.477 | 3.591 | 3.392 | 3.962 |
|  | .960 | 4663.090 | 2836.336 | 11899.664 | 3.669 | 3.453 | 4.076 |
|  | .970 | 5811.554 | 3363.626 | 16432.732 | 3.764 | 3.527 | 4.216 |
|  | .980 | 7787.542 | 4213.998 | 25269.572 | 3.891 | 3.625 | 4.403 |
|  | .990 | 12352.035 | 5999.476 | 49890.676 | 4.092 | 3.778 | 4.698 |
| a. Logarithm base = 10. | | | | | | | |


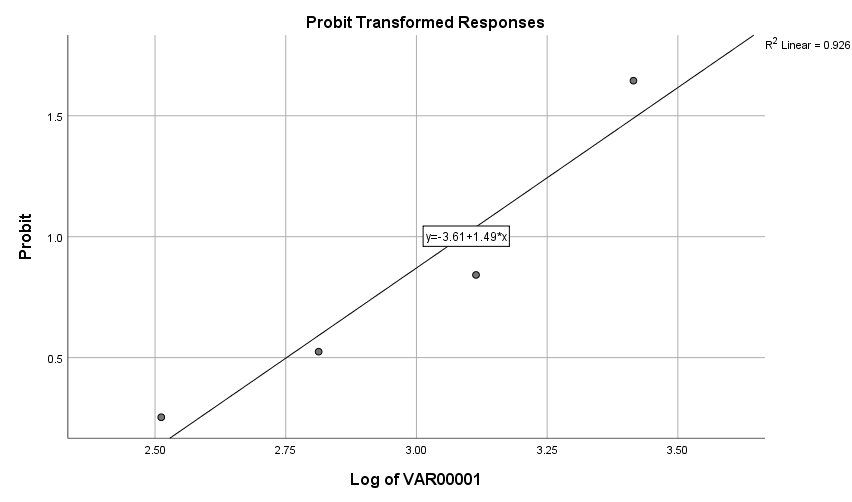


**Probit Analysis 17/34**

| **Data Information** | | |
| --- | --- | --- |
|  | | N of Cases |
| Valid | | 4 |
| Rejected | Missing | 0 |
|  | LOG Transform Cannot be Done | 0 |
|  | Number of Responses > Number of Subjects | 0 |
| Control Group | | 1 |

| **Convergence Information** | | |
| --- | --- | --- |
|  | Number of Iterations | Optimal Solution Found |
| PROBIT | 10 | Yes |

| **Parameter Estimates** | | | | | | | |
| --- | --- | --- | --- | --- | --- | --- | --- |
|  | Parameter | Estimate | Std. Error | Z | Sig. | 95% Confidence Interval | |
|  |  |  |  |  |  | Lower Bound | Upper Bound |
| PROBIT^a^ | VAR00001 | 1.452 | .215 | 6.754 | .000 | 1.031 | 1.873 |
|  | Intercept | -4.892 | .656 | -7.461 | .000 | -5.548 | -4.236 |
| a. PROBIT model: PROBIT(p) = Intercept + BX (Covariates X are transformed using the base 10.000 logarithm.) | | | | | | | |

| **Chi-Square Tests** | | | | |
| --- | --- | --- | --- | --- |
|  | | Chi-Square | df^b^ | Sig. |
| PROBIT | Pearson Goodness-of-Fit Test | 1.249 | 2 | .536^a^ |
| a. Since the significance level is greater than .150, no heterogeneity factor is used in the calculation of confidence limits. | | | | |
| b. Statistics based on individual cases differ from statistics based on aggregated cases. | | | | |

| **Cell Counts and Residuals** | | | | | | | |
| --- | --- | --- | --- | --- | --- | --- | --- |
|  | Number | VAR00001 | Number of Subjects | Observed Responses | Expected Responses | Residual | Probability |
| PROBIT | 1 | 2.512 | 100 | 10 | 10.665 | -.665 | .107 |
|  | 2 | 2.813 | 100 | 20 | 20.971 | -.971 | .210 |
|  | 3 | 3.114 | 100 | 40 | 35.557 | 4.443 | .356 |
|  | 4 | 3.415 | 100 | 50 | 52.663 | -2.663 | .527 |

| **Confidence Limits** | | | | | | | |
| --- | --- | --- | --- | --- | --- | --- | --- |
|  | Probability | 95% Confidence Limits for VAR00001 | | | 95% Confidence Limits for log(VAR00001)^a^ | | |
|  |  | Estimate | Lower Bound | Upper Bound | Estimate | Lower Bound | Upper Bound |
| PROBIT | .010 | 58.465 | 17.283 | 115.699 | 1.767 | 1.238 | 2.063 |
|  | .020 | 90.079 | 31.633 | 162.477 | 1.955 | 1.500 | 2.211 |
|  | .030 | 118.504 | 46.380 | 201.708 | 2.074 | 1.666 | 2.305 |
|  | .040 | 145.658 | 61.812 | 237.494 | 2.163 | 1.791 | 2.376 |
|  | .050 | 172.273 | 78.041 | 271.376 | 2.236 | 1.892 | 2.434 |
|  | .060 | 198.724 | 95.127 | 304.138 | 2.298 | 1.978 | 2.483 |
|  | .070 | 225.239 | 113.110 | 336.246 | 2.353 | 2.054 | 2.527 |
|  | .080 | 251.968 | 132.025 | 368.014 | 2.401 | 2.121 | 2.566 |
|  | .090 | 279.021 | 151.897 | 399.668 | 2.446 | 2.182 | 2.602 |
|  | .100 | 306.485 | 172.753 | 431.383 | 2.486 | 2.237 | 2.635 |
|  | .150 | 452.077 | 292.504 | 595.397 | 2.655 | 2.466 | 2.775 |
|  | .200 | 615.704 | 439.214 | 778.491 | 2.789 | 2.643 | 2.891 |
|  | .250 | 802.545 | 612.453 | 995.901 | 2.904 | 2.787 | 2.998 |
|  | .300 | 1018.193 | 808.707 | 1268.314 | 3.008 | 2.908 | 3.103 |
|  | .350 | 1269.432 | 1023.702 | 1621.869 | 3.104 | 3.010 | 3.210 |
|  | .400 | 1564.929 | 1256.993 | 2086.122 | 3.194 | 3.099 | 3.319 |
|  | .450 | 1916.139 | 1513.372 | 2696.260 | 3.282 | 3.180 | 3.431 |
|  | .500 | 2338.650 | 1801.297 | 3500.357 | 3.369 | 3.256 | 3.544 |
|  | .550 | 2854.326 | 2132.126 | 4569.565 | 3.456 | 3.329 | 3.660 |
|  | .600 | 3494.910 | 2521.085 | 6013.606 | 3.543 | 3.402 | 3.779 |
|  | .650 | 4308.450 | 2989.808 | 8008.709 | 3.634 | 3.476 | 3.904 |
|  | .700 | 5371.558 | 3571.174 | 10853.408 | 3.730 | 3.553 | 4.036 |
|  | .750 | 6814.926 | 4319.040 | 15091.029 | 3.833 | 3.635 | 4.179 |
|  | .800 | 8882.972 | 5330.182 | 21813.420 | 3.949 | 3.727 | 4.339 |
|  | .850 | 12098.127 | 6802.584 | 33556.944 | 4.083 | 3.833 | 4.526 |
|  | .900 | 17845.218 | 9233.856 | 57772.645 | 4.252 | 3.965 | 4.762 |
|  | .910 | 19601.670 | 9939.500 | 65884.111 | 4.292 | 3.997 | 4.819 |
|  | .920 | 21706.283 | 10766.690 | 75996.360 | 4.337 | 4.032 | 4.881 |
|  | .930 | 24282.160 | 11755.144 | 88921.458 | 4.385 | 4.070 | 4.949 |
|  | .940 | 27521.978 | 12965.803 | 105979.552 | 4.440 | 4.113 | 5.025 |
|  | .950 | 31747.856 | 14498.274 | 129473.934 | 4.502 | 4.161 | 5.112 |
|  | .960 | 37548.902 | 16530.046 | 163829.765 | 4.575 | 4.218 | 5.214 |
|  | .970 | 46152.734 | 19419.620 | 218828.179 | 4.664 | 4.288 | 5.340 |
|  | .980 | 60716.296 | 24052.983 | 321583.404 | 4.783 | 4.381 | 5.507 |
|  | .990 | 93547.885 | 33688.861 | 590140.403 | 4.971 | 4.527 | 5.771 |
| a. Logarithm base = 10. | | | | | | | |


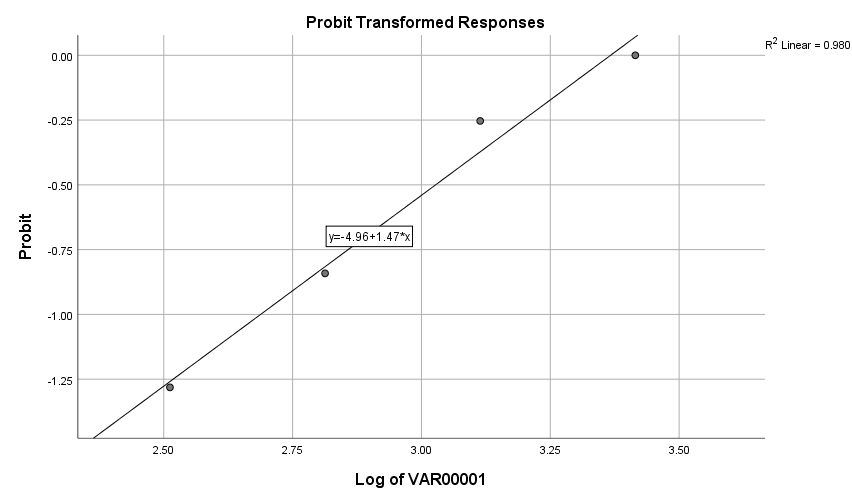


**Probit Analysis**

**Probit Analysis 17/35**

| **Data Information** | | |
| --- | --- | --- |
|  | | N of Cases |
| Valid | | 4 |
| Rejected | Missing | 0 |
|  | LOG Transform Cannot be Done | 0 |
|  | Number of Responses > Number of Subjects | 0 |
| Control Group | | 1 |

| **Convergence Information** | | |
| --- | --- | --- |
|  | Number of Iterations | Optimal Solution Found |
| PROBIT | 10 | Yes |

| **Parameter Estimates** | | | | | | | |
| --- | --- | --- | --- | --- | --- | --- | --- |
|  | Parameter | Estimate | Std. Error | Z | Sig. | 95% Confidence Interval | |
|  |  |  |  |  |  | Lower Bound | Upper Bound |
| PROBIT^a^ | VAR00001 | 1.433 | .201 | 7.132 | .000 | 1.039 | 1.827 |
|  | Intercept | -4.387 | .602 | -7.285 | .000 | -4.989 | -3.785 |
| a. PROBIT model: PROBIT(p) = Intercept + BX (Covariates X are transformed using the base 10.000 logarithm.) | | | | | | | |

| **Chi-Square Tests** | | | | |
| --- | --- | --- | --- | --- |
|  | | Chi-Square | df^b^ | Sig. |
| PROBIT | Pearson Goodness-of-Fit Test | 1.185 | 2 | .553^a^ |
| a. Since the significance level is greater than .150, no heterogeneity factor is used in the calculation of confidence limits. | | | | |
| b. Statistics based on individual cases differ from statistics based on aggregated cases. | | | | |

| **Cell Counts and Residuals** | | | | | | | |
| --- | --- | --- | --- | --- | --- | --- | --- |
|  | Number | VAR00001 | Number of Subjects | Observed Responses | Expected Responses | Residual | Probability |
| PROBIT | 1 | 2.512 | 100 | 20 | 21.538 | -1.538 | .215 |
|  | 2 | 2.813 | 100 | 40 | 36.073 | 3.927 | .361 |
|  | 3 | 3.114 | 100 | 50 | 52.983 | -2.983 | .530 |
|  | 4 | 3.415 | 100 | 70 | 69.364 | .636 | .694 |

| **Confidence Limits** | | | | | | | |
| --- | --- | --- | --- | --- | --- | --- | --- |
|  | Probability | 95% Confidence Limits for VAR00001 | | | 95% Confidence Limits for log(VAR00001)^a^ | | |
|  |  | Estimate | Lower Bound | Upper Bound | Estimate | Lower Bound | Upper Bound |
| PROBIT | .010 | 27.430 | 6.993 | 60.247 | 1.438 | .845 | 1.780 |
|  | .020 | 42.508 | 12.756 | 85.203 | 1.628 | 1.106 | 1.930 |
|  | .030 | 56.127 | 18.669 | 106.207 | 1.749 | 1.271 | 2.026 |
|  | .040 | 69.178 | 24.856 | 125.394 | 1.840 | 1.395 | 2.098 |
|  | .050 | 82.002 | 31.365 | 143.565 | 1.914 | 1.496 | 2.157 |
|  | .060 | 94.774 | 38.223 | 161.126 | 1.977 | 1.582 | 2.207 |
|  | .070 | 107.599 | 45.451 | 178.316 | 2.032 | 1.658 | 2.251 |
|  | .080 | 120.549 | 53.066 | 195.292 | 2.081 | 1.725 | 2.291 |
|  | .090 | 133.674 | 61.084 | 212.165 | 2.126 | 1.786 | 2.327 |
|  | .100 | 147.015 | 69.521 | 229.019 | 2.167 | 1.842 | 2.360 |
|  | .150 | 217.983 | 118.521 | 314.961 | 2.338 | 2.074 | 2.498 |
|  | .200 | 298.109 | 180.413 | 407.289 | 2.474 | 2.256 | 2.610 |
|  | .250 | 389.952 | 257.492 | 510.191 | 2.591 | 2.411 | 2.708 |
|  | .300 | 496.311 | 352.185 | 628.524 | 2.696 | 2.547 | 2.798 |
|  | .350 | 620.602 | 466.663 | 769.246 | 2.793 | 2.669 | 2.886 |
|  | .400 | 767.208 | 602.241 | 943.072 | 2.885 | 2.780 | 2.975 |
|  | .450 | 941.935 | 759.137 | 1166.188 | 2.974 | 2.880 | 3.067 |
|  | .500 | 1152.699 | 937.715 | 1461.286 | 3.062 | 2.972 | 3.165 |
|  | .550 | 1410.623 | 1141.035 | 1858.763 | 3.149 | 3.057 | 3.269 |
|  | .600 | 1731.883 | 1376.635 | 2401.472 | 3.239 | 3.139 | 3.380 |
|  | .650 | 2141.009 | 1657.361 | 3155.919 | 3.331 | 3.219 | 3.499 |
|  | .700 | 2677.183 | 2003.337 | 4234.139 | 3.428 | 3.302 | 3.627 |
|  | .750 | 3407.379 | 2447.382 | 5840.021 | 3.532 | 3.389 | 3.766 |
|  | .800 | 4457.138 | 3048.203 | 8382.988 | 3.649 | 3.484 | 3.923 |
|  | .850 | 6095.498 | 3925.862 | 12812.243 | 3.785 | 3.594 | 4.108 |
|  | .900 | 9037.935 | 5383.038 | 21907.847 | 3.956 | 3.731 | 4.341 |
|  | .910 | 9939.978 | 5807.689 | 24946.303 | 3.997 | 3.764 | 4.397 |
|  | .920 | 11022.239 | 6306.384 | 28729.691 | 4.042 | 3.800 | 4.458 |
|  | .930 | 12348.741 | 6903.500 | 33559.130 | 4.092 | 3.839 | 4.526 |
|  | .940 | 14019.807 | 7636.501 | 39923.575 | 4.147 | 3.883 | 4.601 |
|  | .950 | 16203.394 | 8566.748 | 48675.014 | 4.210 | 3.933 | 4.687 |
|  | .960 | 19207.154 | 9803.828 | 61447.874 | 4.283 | 3.991 | 4.789 |
|  | .970 | 23673.413 | 11569.699 | 81848.402 | 4.374 | 4.063 | 4.913 |
|  | .980 | 31257.971 | 14414.840 | 119850.968 | 4.495 | 4.159 | 5.079 |
|  | .990 | 48439.403 | 20374.402 | 218737.151 | 4.685 | 4.309 | 5.340 |
| a. Logarithm base = 10. | | | | | | | |


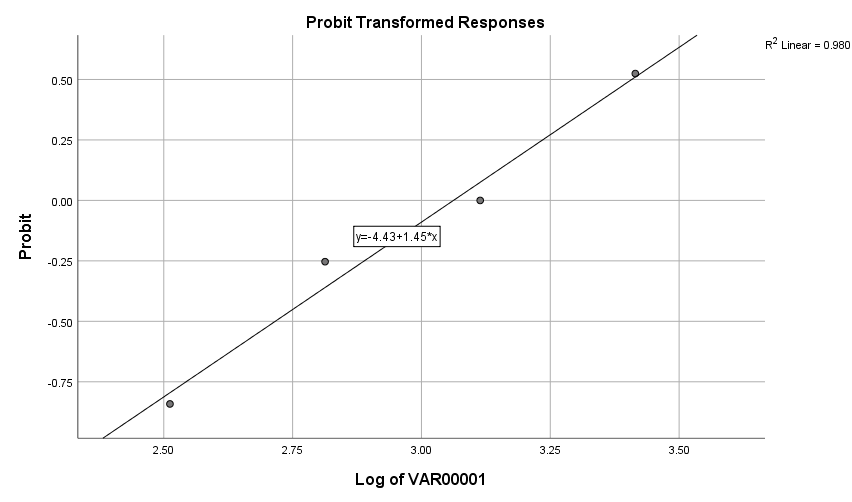

Supplement: S1 Text — (DOCX) [file pone.0221382.s002.docx]
