# Supplementary material for: Growth behavior and glyphosate resistance level in 10 populations of Echinochloa colona in Australia
Source: PLoS One. 2020 Jan 14;15(1):e0221382. doi: 10.1371/journal.pone.0221382 (PMC6959982; doi:10.1371/journal.pone.0221382)
Supplement: Attachment — Submitted filename: Response letter (6).docx [file pone.0221382.s003.docx]

Dr Xiao Guo,
Academic Editor
PLOS ONE

Subject: Ref.:  PONE-D-19-21773: Growth behavior and glyphosate resistance level in 10 biotypes of *Echinochloa colona* in Australia
PLOS ONE

Dear Dr Guo,

We thank you for the critical look on the manuscript and highly appreciate the remarks of the reviewers to improve the manuscript. The manuscript has been revised in the light of useful comments. All the comments have been incorporated in the revised version carefully. For clarity, the comments and suggestions will appear in the black colored text; while our response will appear in the blue text. In the manuscript also, specific changes can be seen in the highlighted text. We hope that the revised manuscript is now acceptable in “PLOS ONE”.

We look forward to hearing from you in due course.

Sincerely yours

Gulshan Mahajan

==============================
ACADEMIC EDITOR:

The comments of three reviewers have been received as presented below. Two of them recommended major revision while another one recommended rejection. Please revise the manuscript according to the comments of the three reviewers. Particularly, Rev.# 1 felt that the definition of herbicide resistance should be demonstrated according to reliable literature. Besides, two reviewers reviewed that statistical results have not been sufficiently presented and discussed. Please address all comments one by one and attach a response letter of a list or explanation of changes made to the manuscript in your revised version.

==============================

We have addressed all comments of three reviewers and attempt has been made to incorporate the critical comments of reviewer#1 carefully.

1. In your Methods section, please provide additional information regarding the permits you obtained for the work. Please ensure you have included the full name of the authority that approved the collection sites access and, if no permits were required, a brief statement explaining why.

Yes, permissions were obtained from the consultants through phones and personal meetings.


Reviewers' comments:

Reviewer #1: Overall, the manuscript is written well and clearly. The subject should be of interest of readers of weed science community.

My biggest question is one of semantics, I believe. I read the criteria for listing a species as resistant on the website for International Survey of Herbicide Resistant Weeds (I. Heap; http://weedscience.com/Documents/ResistanceCriterion.pdf). There are problems with the research methods used that do not support the results presented. The definition of herbicide resistance is based on plant/population survival not plant growth (biomass). Check the WSSA website for the accepted definition of herbicide resistance. In the case of these studies where there was only few plants per pot, population survival cannot be effectively determined and therefore, it is not possible to definitively characterize the resistance status of the collected populations.

We have also recorded the data for survival %. We again reanalysed the data and calculated LD_50_ value on the basis of survival %. We used several doses, so the number of plants used per pots were sufficient. Yes, for only one population, they were not enough.

Once characterized, plant biomass data can be used to indicate the robustness of the resistance mechanism. However, this is only once resistance has been confirmed. The recommendation is that there is a need for further studies aimed at characterizing the resistance status of the collected biotypes based on survival of a reasonable population size (e.g. 40-50 seedlings). A known susceptible biotype needs to be included and replication is not needed if there is a sufficiently large population size. See methods in Owen et al 2016.

There were five seedlings for each biotype in four replications. So 20 seedlings for each biotype. As the experiment was repeated twice; therefore, results are based on 40 seedlings per biotype. We had a susceptible biotype B 17/37. The data has been reanalysed for survival%.

The whole plant dose response experiments appear to provide more definitive evidence of resistance. However, based on the author clarification of experimental design the dose response experiment may not be confirmatory if the study was not set up to statistically compare the putative resistant population with the susceptible population. Does the work also need to demonstrate heritability in order to confirm resistance in this case? I suspect literature evidence may be important since experimental evidence is lacking.

We followed the publication of Gaines et al. 2012 for experimental design and planning that was published in Weed Technology and they did not demonstrate heritability in order to confirm resistance. Yes, we had a susceptible biotype named B 17/37 in these 10 biotypes.

Gaines T.A., Cripps A., Powles S.B. Evolved resistance to glyphosate in junglerice (*Echinochloa colona*) from the tropical Ord River region in Australia. Weed Technology, 2012 26: p. 480-484.

SPECIFIC COMMENTS
L 97: Please describe results and discussion in two separate sections.

Results and discussion are combined here according to the format of the journal. Moreover, combined results and discussion is good to avoid repetition. However at the end, we have concluded our results in a general discussion.

L 251-254: How many plants per site were collected? What was the area occupied by those plants per location? Was there a protocol for collecting seed that defined the area of collection? How large a seed sample per population was obtained? Were sample sizes similar among populations? What was the germination percent for the sampled populations and did seed viability/germination rate stay constant for the two runs? The authors might consider including the designated names of collected biotypes.

There were 40-50 plants per site and the area occupied by those plants per location was >1 ha. Seeds were collected directly from plants by shaking. Only matured plants were chosen for collection of seeds. There were about 10, 000 seeds per population. The sample size was different depending on infestation levels. The germination rate (seed viability) was >80% for each biotype in both the experimental runs. The collected biotypes were designated on the basis of year and serial number in which these were collected for example 17/37 was designated as sample was collected in 2017 and 37 was the serial number of that biotype in collection.

L 285-287: How did you come up with 650 g/ha rate of glyphosate as 1X recommended rate? This is a low rate of glyphosate to go with. Who recommended this rate and for what situation? If this was based on chemical fallow, don’t you think 1260 g/ha rate will be much appropriate to go with. No adjuvant was included in those glyphosate doses? Dec 5, 2018 to Jan 14, 2018?

The commercial product of glyphosate named Glymount containing active constituent 450 g/L was used and under fallow condition in Australia, its labelled rate is 0.8 -1.2 L/ha

<https://growchoice.com.au/wp-content/uploads/2015/05/Glymount-DFU.pdf>.

Therefore, this is not a low rate of glyphosate in Australia. No adjuvant was included in glyphosate doses. We have corrected the date- it is Jan14, 2019.

L 307-314: The section on statistical analyses is not complete with regard to the glyphosate dose-response analysis. How did you run the probit analysis? Provide the equation used and explained it here. How did you calculate the resistance index when there was no known susceptible biotype included?

Probit analysis was done using software IBM SPSS Statistics 20.0 (SPSS, Inc., Chicago, IL, USA). The equation has been provided and explained. Resistance index was calculated on the basis of susceptible biotype named B 17/37 on a factor basis. Equation (response curve) has been now provided for each biotype. The equation used for estimating response is as:

y = [Intercept + bx (covariate x are transformed using the base 10.0 logarithm)]

Figure 2: Are those GR50 values or Resistant/susceptible factor significantly different from each other? If yes, What statistical test did you perform to find this out? Please explain in the figure legend.

In the revised version, we have now calculated LD50 value and these values were compared using confidence interval overlap as described in table 3. The probit analysis detail of each biotype has been provided in the supplementary file (SPSS software) .

I don’t see any depository for the raw data on these experiments. Please provide all your raw data from where you build these tables and figures. This is the most important requirement of the journal.

We have now provided all raw data on these experiments as supplementary file .

Reviewer #2: The topic is important. The whole paper is clear and well- structured. The experiment is reasonably designed. The result is mostly clear and fully described but I personally think that the result is probably not very well-dug. More analysis about the correlations among variables, the effect comparison of glyphosate usage could be conducted.

1. Please make sure with the journal whether the material and method parts are at the end of the paper or after introduction.

Yes, the material and method parts are at the end of the paper as per journal style.

2. I might miss some information, but it is better having more descriptions about the codes of the species, for example, B17/34, does it have some relationship with the sampling (Fig.1)? It is not so clear and very easy to get confused with these codes when reading.

For B17/34 indicates B is for biotype; 17 indicates sample was collected in 2017 and 34 is the serial number in collection. We reported the biotypes as per our designation in the weed library.

3. The Fig.1 seems not so clear and short of legend and north arrow. The color you chose is too similar with each other which makes the whole figure looks not so good.

The figure 1 has been modified with clear legend and north arrow.

4. There are correlations among leaf, tiller, amount of seeds, etc. Tables are good, but if you have several scatter plots to show these relationships, it might be clearer.

Many correlation between different parameters were significant, therefore, to avoid the confusion, we preferred to show this presentation in table form.

5. For the influences of height, leaf, etc. to the final tiller of the species, there might be interactive effects among different x variables. Is it better you use the multi- correlation analysis, i.e., y = ax1+bx2+cx3…? This is just a suggestion and you could think about this.

We have correlation matrix in table 2 and critical differences were observed at 5% level of significance.

6. Line 134~135, it is not clear using “target” here.

We have replaced this with ‘restrict high’

7. I am not sure, for the unit, you use number plant-1 etc., and do you think plant-1 here is proper? Is it better just delete it and make some explanations in the text? Because all unit is measured by one individual. Or you just use stem-1or individual-1?

It is the standard trend that we have followed and easy to understand.

8. In table 2, delete the last number “1”.

Deleted

9. For table 3, make clear the meaning of each column at the title. “Probit analysis detail” is not clear.

Added. However, all detail of probit analysis has been provided in supplementary file.
10. Line 275, “by shaking” is not precise, you might find another word to describe this operation.

We feel that shaking best describes our method and have used this word in our previous papers to describe this.

11. Line 305~306, more explanation might be needed for the “Fisher’s protected LSD”? At least give the full name of LSD when it is firstly mentioned.

Full name of LSD (least significant differences) has been mentioned now.

12. Is the different doses of the glyphosate used well compared? It is not clear in the result.

LD_50_ values have now been calculated for each biotype and the values compared with a 95% confidence interval.

13. For reference 6 and 7, there are italic styles, make sure with journal and be consist with the others.

Italics have been removed.

14. You declared that the data is available in the supporting information but I did not see any materials attached, please explain this.

We have attached all raw data now.

Reviewer #3: Two experiments were carried out in this study to compare the growth behavior and glyphosate resistance among 10 biotypes of Echinochloa colona in Australia. The growth experiment showed that characteristics like tall nature and high tillering capacity allow E. colona to produce a high leaf number that result in a large number of seed heads and seeds. The glyphosate dose-response experiment showed a wide range of GR50 value in these biotypes, and the biotype B17/34 showed the strongest resistance to glyphosate. The two experiments were conducted rigorously with appropriate replication and sample sizes, and the conclusions were drawn appropriately based on the data presented. In addition, the language of this manuscript is well written and clear.

Thank you for your comments.

The analysis of variance (ANOVA) and Fisher’s protected LSD were applied to test the difference of growth behavior of E. colona among 10 biotypes. And the probit anaylsis were applied to estimate the GR50 value. These statistical methods were performed appropriately, but the outcome of these statistical results have not been sufficiently presented.
Moreover, there are several major and specific comments on this manuscript:

1. The introduction can be reduced to 3 or 4 paragraphs to make the background and aim clearer. The outline of the introduction can refer to:
1) The Echinochloa colona as a noxious weed emerged in Australia crops.
2) The biological characteristics of growth and reproduction of E. colona.
3) The glyphosate is widely used in Australia to eliminate the E. colona, but induced a variety of glyphosate resistant biotypes.
4）the aim and scientific questions of this study

All your points have been addressed and the introduction has been reduced to 4 paragraphs. Differences among growth behaviour of biotypes were compared with LSD values.

2. The author combined the “results” and “discussion” in the manuscript. Although this format can be accepted by some journals, but I suggest divided this part into “results” and “discussions” separately. In the present manuscript, the result of two experiment were discussed respectively, and I can not find their relationships. If there is an independent section of discussion, the relationships between the two experiment and their implications can be better integrated.

Results and discussion are combined here according to the format of the journal and we feel combining the results and discussion avoids repetition. The relationship between the two experiments and the implications has been discussed in general discussion at the end of the article (Conclusion section).


3. The author proposed “growth behavior and seed production potential in these biotypes had no correlation with the resistance index” (LINE 240-241). However, the statistical methods for analyzing the correlation between growth traits and resistance index were not given in the section of “statistical analysis”. Also, I can not find the relevant result which support this conclusion in the context.

Statistical methods for analysing the correlation has been provided under heading statistical analysis. The relationships between parameters were assessed using linear regression analysis. Suggested lines have been added in the conclusion section. The analysis detail of correlation has been provided in the supplementary file.

4. The seed of 10 biotypes of E. colona were collected from a large area in northern grain region in Australia. So, do the geographical differences effect on the growth behavior and glyphosate resistance among 10 biotypes of E. colona. I suggest the multivariate analysis such as PCA or RDA can be applied to study whether the biotypes from closer sites have similar characteristics. Moreover, the correlation (or non-correlation) between growth behavior and resistance index may also be revealed by the multivariate analysis.

We tested these biotypes under one location. Therefore, it is not justified to run PCA analysis. There was no such trend that close sites have similar characteristics. We are studying the genetic diversity of these biotypes in a different experiment.

Here are also several specific comments:
5. Figure 1. It’s better to give a small-scale map of Australia, and pointed out the location of northern grain region.

We have modified figure 1 to incorporate these suggestions.

6. Table 1. the standard deviation of these variables should be given. And as the LSD was performed, I suggest the author add lowercase superscript letters after the values to indicate significance at the 0.05 level for the differences among biotypes.

Letters are to be used in DMRT (Duncan multiple range test) design. LSD values have been given to identify the differences between biotypes. If the difference between two values is more than LSD value, it means the treatments are significantly different. However, we have included the standard deviation in the table.

7. LINE 64: “shikmate pathway” might be “shikimate pathway”

Corrected.

8. Line 251-254: it better to give more information on the seed collected location or the farm, like the crop type, soil type, glyphosate dose and so on, which can help readers know the growth environment of these biotypes.

We have provided the GPS coordinates on map.

However, we are including all information here for your information

| Biotype | Coordinates | Location/Place | Crop |
| --- | --- | --- | --- |
| B17/7 | 27.50000000/151.69666667 | Dalby | Wheat-Fallow |
| B17/12 | 30.268508/149.80481 | Narrabri | Wheat- Fallow |
| B17/13 | 30.306499/149.811438 | Narrabri | Wheat-Fallow |
| B17/16 | 30.09099349/149.64890 | Narrabri | Lathyrus-wheat/chickpea |
| B17/17 | 30.38230/149.59679 | Narrabri | Fallow |
| B17/25 | 28.31500000/148.68916667 | St George | Canal side |
| B17/34 | 28.58305556/150.36888889 | Moree | Wheat fallow |
| B17/35 | 29.95805/149.8339 | Moree | Cotton fallow |
| B17/37 | 27.5514/152.3428 | Gatton | Wheat-Fallow |
| B17/49 | 27.5514/152.3428 | Gatton | Wheat-Fallow |

9. Line 252: how to identify different biotypes? Do they just collected from different sites，or have distinct heritable characteristics?

They were collected from different sites. Heritable characteristics were not studied in this experiment.

10. Line 301: I did not find the ANOVA result

ANOVA results have been provided as annexure.

**Table 1.** ANOVA for different parameters

| **Source** | **Degree of freedom** | **Plant height (cm)** | **Tiller (number plant^-1^)** | **Leaf (number plant^-1^)** | **Seed head (number plant^-1^)** | **Seed head weight (g plant^-1^ )** | **Shoot biomass (g plant^-1^)** | **Root biomass (g plant^-1^)** | **Days to seed head initiation (d)** | **Seed production (number plant^-1^)** |
| --- | --- | --- | --- | --- | --- | --- | --- | --- | --- | --- |
|  |  | **Mean square** | | | | | | | | |
| Replication | 3 | 59.9 | 52.2 | 702.9 | 129.3 | 0.179 | 5.78 | 5.863 | 8.91 | 1621914 |
| Experimental run | 1 | 17155.2* | 9812.4* | 64139.5* | 7940.1* | 202.1* | 15401.2* | 315.96* | 214.5* | 0.433155* |
| Biotype | 9 | 202.8* | 323.5* | 4340.5* | 582.4* | 12.5* | 39.761* | 50.14 | 43.0* | 32654110* |
| Experimental run x Biotype | 9 | 48.8 | 60.9 | 962.2 | 119.9 | 2.59 | 24.083 | 47.84 | 22.5 | 1001201 |
| Error | 57 | 32.0 | 44.1 | 925.5 | 200.9 | 2.75 | 14.584 | 24.5 | 7.34 | 5510769 |

* indicates significant at 5% level

11. Line 307-308 Which variable did the “GR50” in this study based on? The shoot biomass, tiller number, or individuals?

It was based on biomass. However, reviewer# 1 suggested to do on survival % basis. Therefore, now we have provided on survival % basis.

6. PLOS authors have the option to publish the peer review history of their article ([what does this mean?](https://journals.plos.org/plosone/s/editorial-and-peer-review-process#loc-peer-review-history)). If published, this will include your full peer review and any attached files.


If you choose “no”, your identity will remain anonymous but your review may still be made public.

Yes

**Do you want your identity to be public for this peer review?** For information about this choice, including consent withdrawal, please see our [Privacy Policy](https://www.plos.org/privacy-policy).

Reviewer #1: No

Reviewer #2: Yes: Liping Li

Reviewer #3: No

We are okay with Reviewer#2.
