# Supplementary material for: Growth behavior and glyphosate resistance level in 10 populations of Echinochloa colona in Australia
Source: PLoS One. 2020 Jan 14;15(1):e0221382. doi: 10.1371/journal.pone.0221382 (PMC6959982; doi:10.1371/journal.pone.0221382)
Supplement: Attachment — Submitted filename: Response letter (7).docx [file pone.0221382.s004.docx]

Dr Xiao Guo,
Academic Editor
PLOS ONE

Subject: Ref.:  PONE-D-19-21773R1 Growth behavior and glyphosate resistance level in 10 biotypes of *Echinochloa colona* in Australia
PLOS ONE

Dear Dr Guo,

We thank you again for the critical look on the manuscript and highly appreciate the remarks of the reviewers for further improvement of the manuscript. The manuscript has been revised in the light of useful comments. All the comments have been incorporated in the revised version carefully. For clarity, the comments and suggestions will appear in the black colored text; while our response will appear in the blue text. In the manuscript also, specific changes can be seen in the highlighted text. We hope that the revised manuscript is now acceptable in “PLOS ONE”.

We look forward to hearing from you in due course.

Sincerely yours

Gulshan Mahajan

==============================
ACADEMIC EDITOR:

The manuscript has been greatly improved. However, there is still plenty of questions to answer and comments to address, as has been pointed out by reviewer #3. Besides, reviewer #1 asked that “Is there any plan to relate these phenotypic response back to management, cropping systems, location, and/or soils?” in the comments. But I did not find any response to this comment. Please response to this point in the next version.

==============================
This study suggested that glyphosate resistant populations such as B17/34 that produced a high seed number are very problematic. Attempt should be made to reduce its invasion/further infestation. Movement of seeds from one field to another field via various mode of transportation should be restricted. For management of resistant population, crop competition tactics (narrow row spacing, competitive cultivars, optimum seeding rate, cover crops etc.) could be explored in an integrated weed management program to reduce the seed number of resistant populations. The related information has been added in the manuscript.

 Reviewer #2: Personally, I currently do not work a lot on spv files. So I think the supplemental materials are better in xlsx, or txt format. And it is better to have a list of the supporting information.

Agreed. The supplemental material of probit analysis has been attached in a Word file now.

Reviewer #3: The manuscript PONE-D-19-21773 has been improved since the previous version. However, several problems have not yet been solved in the manuscript, although some of them were replied in the response letter. For example, I can see the ANOVA table and site information in the response letter, but can not find them neither in the manuscript nor in the supporting files. Moreover, the ANOVA statistics for the growth characteristics need to be reanalyzed, because the replication was set as a treatment, which I can not agree with. Another problem is, I think there needs some evidence or reference to prove that, the seeds being collected from one site are belong to the same biotype.

The data have been again reanalysed with eight replications as per suggestions. An ANOVA table and site information has been added in the manuscript. The term biotype has been replaced with population.

1. Line 248-250: By definition, the “biotypes” is a group of organisms having the same specific genotype. So, plants with same biotype must have common heritable traits, by which the biotype can be distinguished. In this study, the seeds of one biotype were collected from 40-50 plants per site and over an area >1ha. I wonder whether the seeds of same biotype were collected from one clone, or distinguished by any trait during the seed collection. Otherwise they can only be call “population” but not “biotype”. Since the experimental evidence for heritability traits is lacking, it is better to provide references in discussing this point.

As seeds were not collected from one clone. Therefore, to avoid confusion, we have replaced the term biotype with population.

2. Line 296, “14 January 2018” should be “14 January 2019”

Corrected.

3. Line 312-314, the author supplied the ANOVA table in the response letter, but I can not find it neither in the manuscript nor in the supporting material in xlsx files. Maybe it was included in the SPV format files which I could not open. The SPV file can only be open by SPSS, which is a commercial software and very expensive. I believe that many readers, just like me, have not installed the SPSS software. So, I suggested the contents in SPV files can be transformed into a common format, such as xlsx or txt, which can be opened by all readers.

Moreover, in the ANOVA table, the replicates should not be treated as a factor.

Now we have transferred the content from SPV file to txt file (probit analysis) and the file has been attached as a supplementary file. We have reanalysed the data again with eight replications. An ANOVA table has been added in the text.

4. Line 319, remove “the growth of”

Corrected

5. Figure 1, the author gave a table of the site information in the response letter, but I can neither find it in the manuscript nor supporting information.

Site information from Figure 1 has been added in the manuscript.

6. Table 2, please give the sample size involved in the correlation analysis given in the table caption or note, like “n=?”
Added. n=10.

7. Figure 2 I found the biotypes of B17/7, B17/12, B17/17, B17/25 had no significant difference with the susceptible biotype. Do this mean the five biotypes being susceptible as B17/37? If it is, I suggest separate the all 10 biotypes into two groups, and compare their growth traits between groups. Maybe you can find significant differences of growth characteristics between susceptible and glyphosate resistant biotypes. This is just a suggestion and you could think about this.

Yes, populations B17/7, B17/12, B17/17, B17/25 were susceptible as 17/37. However, they differ in their growth behaviour. We tried to analyse with your suggestion and found these cannot be grouped together on the basis of growth traits.

8. Line 328: Furthermore, do the difference of LD50 and resistance index among the 10 biotypes were compared using LSD method? Please add the information of statistical methods .

No, these were not compared using LSD method. LD_50_ values for each population were compared using confidence interval overlap in probit analysis (Table 4). Resistance index is a factor (resistance/susceptibility ratio); therefore, these were also compared on the basis of LD_50_ value.

9. Line 334, The “funding acquisition” can be removed because there was “no special funding was obtained for this work” in the funding information.

Yes, it has been removed.
